# Mannan immunoassay for diagnosis of invasive mucormycosis: assay development, preclinical testing and evaluation in an animal model

**DOI:** 10.3389/fcimb.2026.1875264

**Published:** 2026-07-22

**Authors:** Amanda R. Burnham-Marusich, Vidhi Pandit, Sondus Alkhazraji, Yiyou Gu, Kathleen R. Zayac, John R. Wingard, Emma L. Culver, Joseph J. Walish, Ashraf S. Ibrahim, Thomas R. Kozel

**Affiliations:** 1 Department of Microbiology and Immunology, University of Nevada, Reno School of Medicine, Reno, NV, United States; 2 The Lundquist Institute, Torrance, CA, United States; 3 Division of Hematology/Oncology, University of Florida College of Medicine, Gainesville, FL, United States; 4 Hyperion Biosystems, Watertown, MA, United States; 5 Division of Infectious Disease, David Geffen School of Medicine, University of California, Los Angeles, Los Angeles, CA, United States

**Keywords:** biomarker, diagnostic, invasive fungal disease, lateral flow immunoassay, mannan, mucormycosis, point of care, rapid test

## Abstract

Early diagnosis of mucormycosis would be greatly facilitated by identification of a non-invasive biomarker for infection. This study evaluated whether detection of Mucorales mannan in serum or bronchoalveolar lavage fluid (BALF) can serve as such a biomarker. To date, mannan has been found in body fluids of invasive disease caused by many ascomycetous fungal pathogens, but its presence has not been evaluated for disease-producing members of the Mucorales. We utilized a monoclonal antibody (mAb) that is reactive with Mucorales mannan to construct a lateral flow immunoassay (LFA) containing labeled europium nanoparticles as the detector reagent. Analytical inclusivity studies found that the LFA detected mannans from four of the five disease-producing Mucorales fungi. No cross-reactivity was observed with purified mannans from other major causes of invasive fungal infection, i.e. *Aspergillus*, *Candida* and *Fusarium*. The LFA was evaluated in a pulmonary mouse model of invasive mucormycosis using serum and BALF specimens. Mannan was detectable in both specimens and increased in concentration through the course of infection. Lastly, an exploratory study for clinical proof-of-concept was performed with a limited set of sera or plasma samples collected at one or more timepoints from patients with probable or proven mucormycosis. Four of the 12 patients had positive test results at one or more times. Treatment with amphotericin B was associated with negative LFA results: among the six patients for whom a specimen was collected while they were not receiving AmB, 4 of the 6 patients had positive test results at one or more times. One patient whose blood was collected prospectively showed a high level of mannan seven days before meeting the EORTC diagnostic criteria. Taken together, the results demonstrate, for the first time, that mannan in blood samples and BALF is a biomarker for infection by the Mucorales fungi and support further study of mannan-targeted diagnostics for mucormycosis.

## 1 Introduction

Mucormycosis is an aggressive opportunistic fungal infection caused by members of the fungal order Mucorales. *Rhizopus* and *Mucor* species account for approximately 65% of cases, with *Cunninghamella*, *Apophysomyces*, *Lichtheimia*, and other genera individually producing 1-7% of the remaining cases (Roden et al., 2005). Invasive mucormycosis occurs most frequently in patients who are immunocompromised, e.g., transplant or hematology-oncology patients or patients with poorly controlled diabetes mellitus. Mucorales fungi are ubiquitous in the environment. Infection typically occurs following inhalation of spores leading to infection of the lung or sinuses. Mucorales fungi also may produce severe soft tissue infections following contamination of wound injuries.

Early diagnosis and rapid initiation of appropriate antifungal therapy are cornerstones for successful treatment of mucormycosis (Walsh et al., 2012). Chamilos et al. found that delayed initiation of amphotericin-B-based therapy of 6 days or more was associated with a 2-fold increase in all-cause mortalities (Chamilos et al., 2008). Definitive diagnosis of mucormycosis is dependent on culturing the fungus from normally sterile locations and histopathology. However, specimens most likely to yield positive results, e.g., pulmonary biopsy, are invasive, have risks, particularly in patients with neutropenia, thrombocytopenia or coagulopathy, and usually yield positive results only in later stages of infection (Walsh et al., 2012; Danion et al., 2015; Kontoyiannis et al., 2012). Detection of β-D-glucan in blood has been effective for diagnosis of invasive aspergillosis or candidiasis but is negative with the Mucorales (Skiada et al., 2013; Walsh et al., 2014). More recently, plasma cell-free DNA PCR has shown great promise for non-invasive diagnosis of mucormycosis (Millon et al., 2013, 2022; Mah et al., 2025; Moreno et al., 2024).

An alternative low-complexity, point-of-care approach that also avoids invasive clinical specimens for culture and histopathology is immunoassay for cell wall mannan shed from the site of infection and detected in serum and urine. Shed mannan has been an effective biomarker for every invasive fungal disease that has been studied to date, e.g., aspergillosis, blastomycosis, candidiasis, fusariosis, histoplasmosis and coccidioidomycosis (Sendid et al., 1999; Reiss and Lehmann, 1979; Stynen et al., 1995; Connolly et al., 2012; Tortorano et al., 2012; Wheat et al., 1986; Durkin et al., 2009). *Aspergillus*, *Candida*, *Fusarium* and all other fungi where mannans are biomarkers in serum and urine are members of the Ascomycota phylum of the fungal kingdom. In contrast, members of the order Mucorales that cause invasive infection are all members of the Mucormycota phylum.

Despite considerable phylogenetic separation, the Ascomycota and the Mucorales share the genetic information needed to encode the α-1,6 mannan that comprises the backbone of most fungal mannans (Burnham-Marusich et al., 2018), suggesting that mannan from the Mucorales may be a biomarker for infection in a manner similar to the invasive genera of the Ascomycetes. The goals of our study were to i) evaluate novel monoclonal antibodies (mAbs) that are reactive with cell wall mannan from clinically important genera of the Mucorales, ii) construct immunoassays in the lateral flow immunoassay (LFA) format for detection of Mucorales mannans, iii) assess the reactivity of a Mucorales mannan immunoassay across mannans from various Mucorales genera and cross reactivity with mannans from other selected Ascomycota genera, iv) determine whether mannans from the Mucorales are shed into serum and BAL fluid in an animal model of mucormycosis, and v) perform proof-of-concept exploratory studies with specimens from mucormycosis patients to determine whether Mucorales mannan is shed into body fluids during infection. The results showed, for the first time, that mannans from the Mucorales can be detected by immunoassay and that shed mannans are a biomarker for infection.

## 2 Methods

### 2.1 Fungal mannan

The fungal strains and sources of fungal cultures used for this study are summarized in**Supplementary Table 1**. All fungi were cultured in Caisson Labs RPMI-1640 supplemented with 2% (w/v) glucose at 37°C on a rotary shaker. Fungal mycelia were harvested from the culture medium by filtration with 0.45-μm filters and then washed extensively over Whatman #1 filter paper with deionized, 0.22-μm-filtered water. *Candida albicans* blastoconidia were harvested by centrifugation at 4,000 × *g* for 5 min and washed with water three times, centrifuging again after each wash. Mannans were extracted by a modification of the hot citrate method of Peat (Peat et al., 1961). Briefly, the washed fungal cells were collected, weighed, and resuspended in 10 volumes (W/V) of 0.1 M citrate buffer, pH 7.0. The cell suspensions were autoclaved for 25 min. The autoclaved cells were harvested by 0.22-μm filtration or, in the case of *Candida* cells, by centrifugation, and the filtrate or supernatant fluid was collected. The washed cells were resuspended in an additional 10 volumes of 0.1 M citrate buffer, pH 7.0 and autoclaved again for 25 minutes, after which the cells were again separated from the supernatant fluid as above, and the filtrate or supernatant fluid was collected. Mannan was purified from the pooled fluids by affinity chromatography on concanavalin A-Sepharose 4B, dialyzed against water, lyophilized, and weighed. Purified mannans were resuspended in water and sterilized by 0.22-μm filtration prior to use.

### 2.2 mAb production

mAb 7C11 was provided by DxDiscovery, Inc. (Reno, NV, USA). The mAb had been prepared from splenocytes from mice immunized with mycelia of *Rhizopus oryzae* 99-892. The immunization and hybridoma production protocols have been reported (Burnham-Marusich et al., 2018). All mAb 7C11 vertebrate animal work was approved by the Institutional Animal Care and Use Committee of the University of Nevada, Reno, which is the IACUC of record for DxDiscovery. Hybridomas were subjected to positive screening with purified mannans from various Mucorales genera and negative screening with mannans from other fungi that might produce invasive fungal infection. Hybridomas of interest were cloned at least twice by limiting dilution to ensure monoclonality. Antibody was purified via Protein A affinity chromatography.

### 2.3 Ouchterlony double immunodiffusion

Rosettes were cut into agarose plates prepared with 1X phosphate buffered saline (PBS), pH 7.4 containing 1% (w/v) agarose. Purified mannans at 0.5 mg/mL in 1X PBS, pH 7.4 were loaded into the outer ring wells of the rosette; mAb 7C11 at 1 mg/mL in 1X PBS, pH 7.4 was loaded into the center well of the rosette. Plates were allowed to incubate overnight at room temperature. Lines of white precipitate, indicating antibody-antigen reactivity, were photographed using darkfield illumination.

### 2.4 Lateral flow immunoassay for detection of mannan

An LFA based on mAb 7C11 was constructed using Estapor^®^ Europium Microspheres as described (Sigma-Aldrich, 2020, 2022). mAb 7C11 was used in the test line. Europium microspheres conjugated to mAb 7C11 were used as the detector reagent in the conjugate pad. Goat anti-mouse IgG was used for the control line (Catalog #7455507, Lampire Biological Laboratories, Pipersville, PA, USA). LFAs were run by placing an LFA test strip into a microtube containing 100 μL of sample. After 15 min, the strip was removed from the tube and quantitative measurement of the LFA test and control line intensities were done with a Halo™ universal fluorescence reader from Hyperion Biosystems (formerly C2Sense, Inc.), Watertown, MA, USA (Walish et al., 2022).

Analytical sensitivity, inclusivity and specificity were done using purified mannans diluted in phosphate buffered saline containing 0.05% (v/v) Tween-20 (PBS-T). BAL fluid from the mouse model was used undiluted in the LFA. Serum/plasma from the mouse model and patients was diluted 1:4 with 0.1M EDTA in 1X PBS-T, pH 8, heated in a boiling water bath for 6 min, clarified by centrifugation (21,130 x *g* for 15 min) and used in the LFA. Limit of blank (LoB) and limit of detection (LoD) were determined according to CLSI guideline EP17 (CLSI/NCCLS, 2004) using GraphPad Prism v11.

### 2.5 Animal model of mucormycosis

A neutropenic mouse model of pulmonary mucormycosis was used as a source of serum and bronchoalveolar lavage fluid (BALF) for evaluation of the diagnostic sensitivity of a Mucorales mannan immunoassay (Luo et al., 2014). Male CD-1 mice were immunosuppressed with intraperitoneal injection of cyclophosphamide (200 mg/kg) and subcutaneous administration of cortisone acetate (500 mg/kg) on day -2 and day +3, relative to infection. Mice were intratracheally infected with approximately 2.5 × 10^5^ spores of *Rhizopus delemar* (99-880). Mice were sacrificed 6, 24, 72 and 120 h after infection and BAL fluid and blood were collected. All mouse infection experiments were approved by the Lundquist Institute IACUC.

### 2.6 Patient specimens

Plasma from patients with mucormycosis were obtained from the University of Florida from the Aspergillosis Technology Consortium (AsTeC) specimen repository (Wingard et al., 2021) (n=10 patients). The repository contained prospectively collected clinical samples and medical information from patients of all ages at high risk for invasive aspergillosis, e.g., bone marrow transplant, hematologic malignancy and lung transplant subjects. Specimens were collected at enrollment and at intervals thereafter. Some of these patients went on to develop symptoms of invasive fungal disease and some did not. AsTeC also enrolled patients from the same high-risk population who already showed symptoms of potential invasive fungal disease. Patients who developed symptoms after enrollment and patients who entered the study with symptoms provided specimens i) at the time fungal infection was first suspected, and ii) at up to 4 additional timepoints thereafter. Patients were classified as having no, possible, probable or confirmed invasive fungal disease using the EORTC/MSG 2008 diagnostic criteria (De Pauw et al., 2008). The day of diagnosis was defined as the first day that all components of the diagnostic criteria were met (Wingard et al., 2021). Although the AsTeC repository was intended for validation of diagnostics for invasive aspergillosis, the patient enrollment criteria led to collection of specimens from patients ultimately found to have mucormycosis. Sera were also prospectively collected across multiple timepoints before and after symptom onset from two patients in the BMT CTN 0101 trial of antifungal prophylaxis who developed mucormycosis (Wingard et al., 2010). These specimens were provided by the University of Florida. Patients in the BMT CTN 0101 Trial were scored using the 2002 EORTC/MSG criteria (Ascioglu et al., 2002). The use of de-identified patient specimens was approved by the University of Nevada, Reno IRB. All patient specimens were barcoded, and LFAs were interpreted by a quantitative electronic fluorescence reader. Plasma and sera from uninfected individuals were used as negative controls. LFA cutoff for each matrix was defined as the mean of the negative sera or plasma plus three standard deviations.

## 3 Results

### 3.1 mAb 7C11 inclusivity and specificity

**Figure 1:**
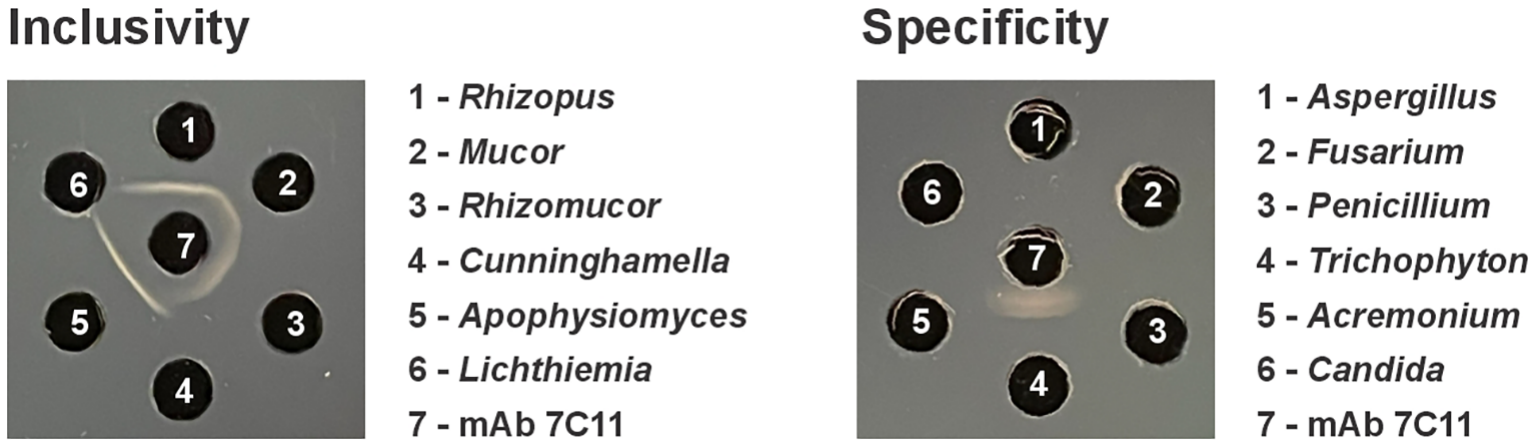
Ouchterlony double immunodiffusion showing reactivity of mAb 7C11 with purified mannans of the Mucorales (inclusivity: *R. oryzae* 46599, *M. circinelloides* 8542 *C. bertholletiae* 175, *A. elegans* 90757, *R. pusillus* 46342, and *L. corymbifera* B2541) and cross-reactivity of mAb 7C11 with purified mannans from other fungi (specificity: *A. fumigatus* 4609, *F. solani* 95-2478, *P. chrysogenum* 10106, *T. rubrum* 4438, *A. strictum* Y-48335, and *C. albicans* 2876).

Efforts to produce mAbs having a high degree of reactivity with mannans from the Mucorales yielded mAb 7C11, which showed broad reactivity across most genera of the Mucorales (inclusivity) and no reactivity with mannans from other fungi that may produce invasive fungal infection (specificity) (**Figure 1**). Specifically, cross-linking of the mAbs with mannans from the Mucorales (inclusivity) and mannans from various Ascomycete fungi (specificity) was evaluated by Ouchterlony double immunodiffusion in agar (**Figure 1**). mAb 7C11 produced precipitin lines with mannans from the Mucorales, including *Rhizopus*, *Mucor*, *Rhizomucor*, *Cunninghamella* and *Apophysomyces*. mAb 7C11 showed no visible precipitin bands with mannan from *Lichthiemia* or the Ascomycete fungi *Aspergillus*, *Fusarium*, *Penicillium*, *Acremonium* or *Candida*. mAb 7C11 was highly reactive with mannan from *Trichophyton*.

### 3.2 mAb 7C11 lateral flow immunoassay analytical performance

**Figure 2:**
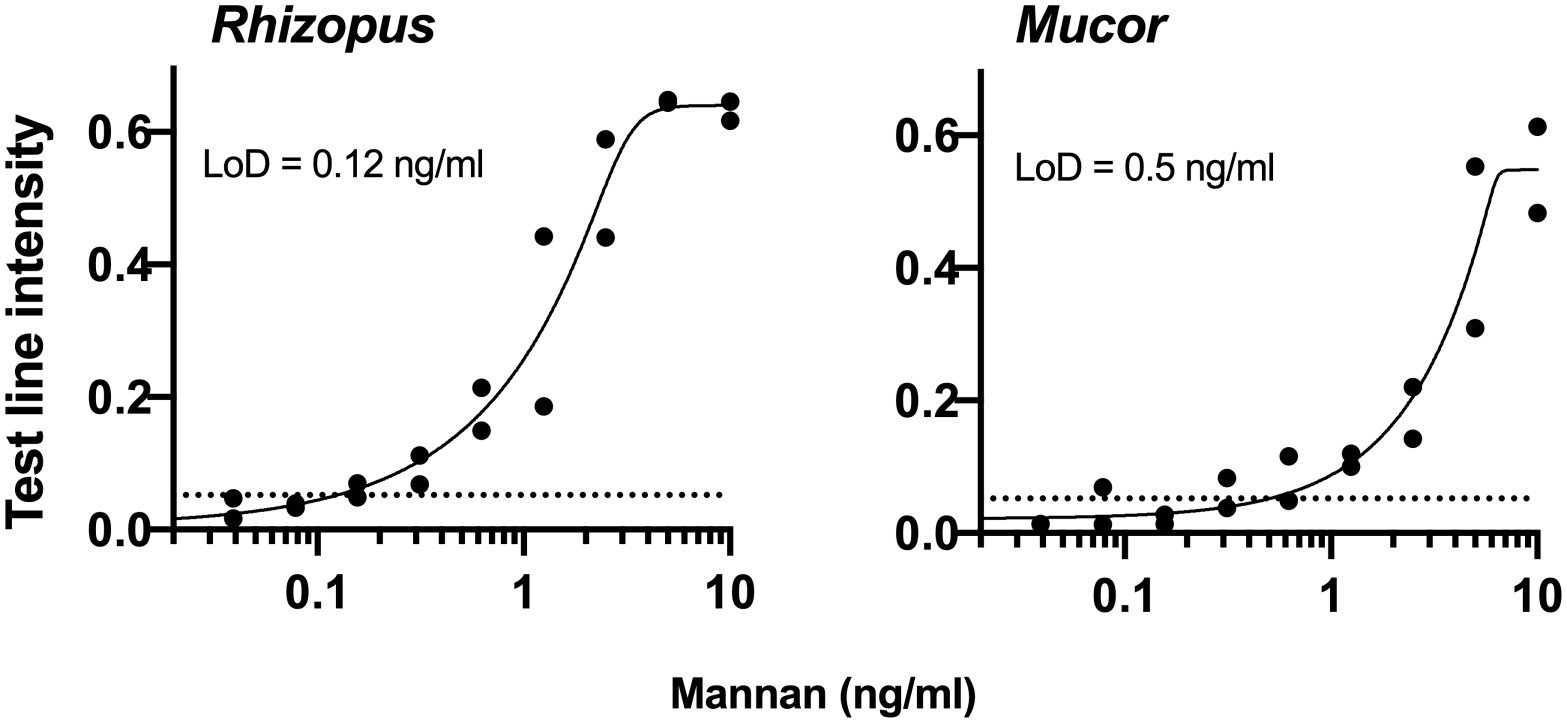
Limit of detection for detection of purified mannan from *R. oryzae* 99892 and *M. circinelloides* 8097. Results are shown from two independent experiments. The limit of detection is shown as a dotted line.

A lateral flow immunoassay (LFA) was constructed using mAb 7C11 in the test line and a europium conjugate of mAb 7C11 as the mobile indicator particle. An initial experiment determined the limit of detection (LoD) of the LFA for detection of purified mannan from isolates of *Rhizopus* and *Mucor*. The results (**Figure 2**) showed LoDs of 0.12 and 0.50 ng/ml respectively for mannans of *Rhizopus* and *Mucor*.

Further experiments were done to evaluate the sensitivity of the LFA for detection of mannans from additional members of the Mucorales (inclusivity) and mannans from various Ascomycete fungi (specificity). The results largely paralleled results obtained by immunodiffusion. The Mucorales LFA detected mannans from species of *Rhizopus*, *Mucor*, *Cunninghamella* and *Apophysomyces* with limits of detection that ranged from 0.12 to 1.2 ng/ml (**Table 1**). There was no reactivity with mannans from *Lichtheimia* spp. The LFA was highly reactive with mannans from *Alternaria* and *Trichophyton* but showed no cross-reactivity with mannans from *Aspergillus*, *Fusarium*, *Penicillium* and *Scedosporium* or various species of *Candida* (**Table 2**).

**Table 1:** Limit of detection for mannans of the Mucorales (inclusivity).

Fungus	LoD (ng/ml) ± 95% CI
*Rhizopus oryzae* 99-892	0.12 (0.05-0.26)
*Rhizopus oryza*e 46599	0.60 (0.19-1.3)
*Rhizopus delemar* 99-880	0.27 (0.13*–0.81)
*Rhizopus microspores* FP-469	0.34 (0.14 – 0.66)
*Rhizopus pusillu*s 46342	0.60 (0.19 – 1.2)
*Rhizopus pusillu*s 32627	1.2 (0.48 – 2.1)
*Mucor circinelloide*s 8097	0.50 (0.09 – 1.2)
*Mucor circinelloide*s 8542	0.62 (0.04* – 1.8)
*Cunninghamella bertholletiae* 174	1.1 (0.61 – 1.5)
*Cunninghamella bertholletiae* 175	0.92 (0.33 – 1.6)
*Apophysomyces elegan*s 90757	0.36 (0.26 – 0.50)
*Lichtheimia corymbifera* B2541	> 10
*Lichtheimia corymbifera* B5792	> 10

*The lower bound of the 95% CI could not be computed for this mannan; the narrower 90% CI lower bound is reported instead.

**Table 2:** Reactivity of mannan from non-Mucorales genera (specificity).

Fungus	LoD (ng/ml) ± 95% CI
*Alternaria alternat*a 6663	0.25 (0.18 – 0.34)
*Aspergillus fumigatu*s 4609	> 10
*Acremonium strictum* Y-48335	> 10
*Candida albican*s 2876	> 10
*Candida auris* CAU-07	> 10
*Candida glabrat*a 48435	> 10
*Candida kruse*i 90878	> 10
*Candida parapsilosi*s 22019	> 10
*Fusarium solani* 95-2478	> 10
*Penicillium chrysogenu*m 10106	> 10
*Scedosporium apiospermu*m 3635	> 10
*Trichophyton rubru*m 4438	0.71 (0.34 – 1.2)

### 3.3 LFA performance with mouse model of invasive mucormycosis

**Figure 3:**
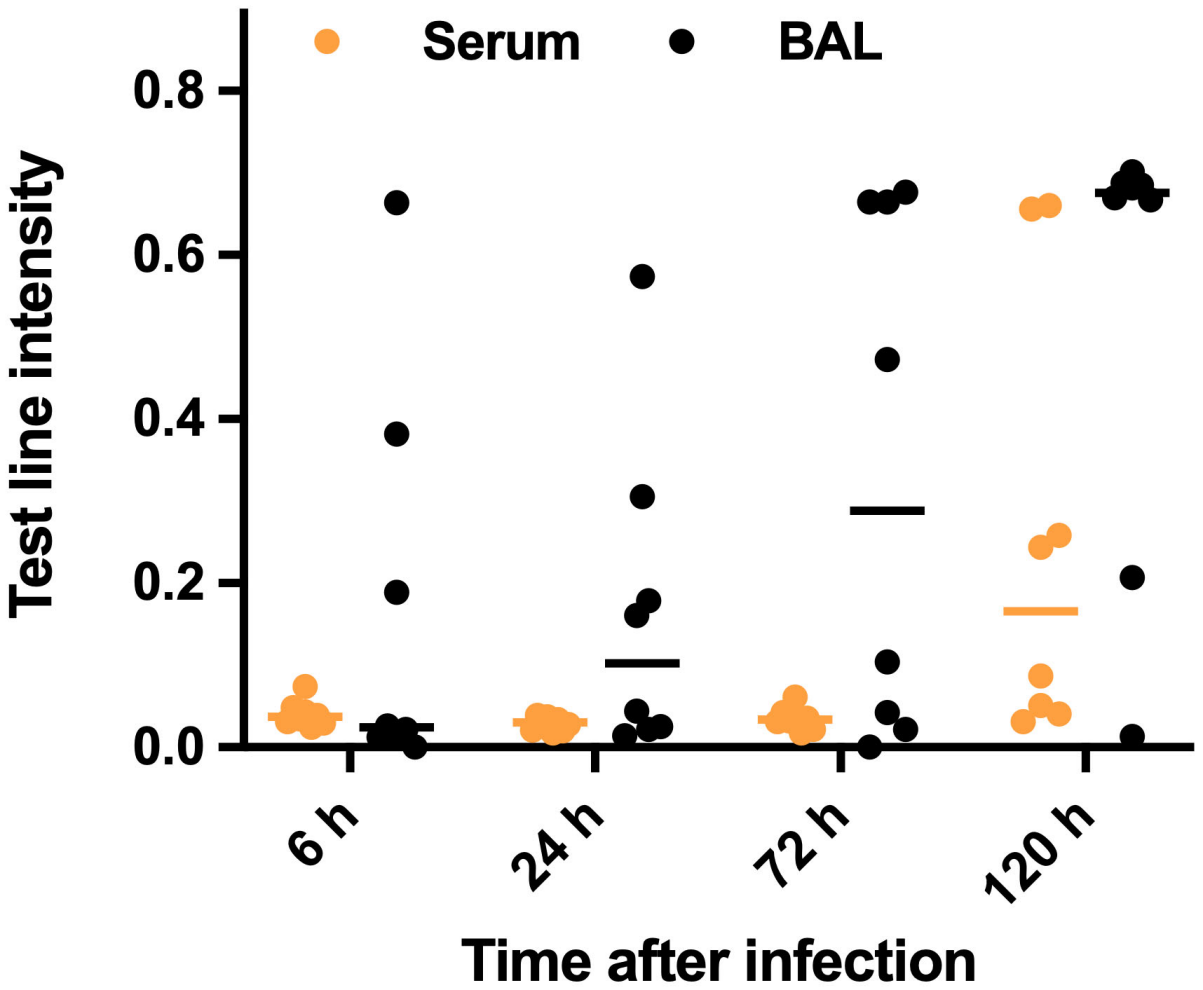
Detection of mannan in serum and BALF in a murine model of infection by *R. delemar* 99-880. Results are shown from individual mice that were sacrificed at the indicated times after pulmonary infection.

The Mucorales LFA was evaluated using samples from a neutropenic mouse model of pulmonary mucormycosis. Mice were infected via the pulmonary route with spores of *R. delemar*. Mice were sacrificed i) prior to infection (negative controls) or ii) 6, 24, 72 or 120 after infection, and BAL fluid and blood were collected. Specimens were processed and evaluated by LFA as described in the Methods. Mannan concentrations increased with time after infection as shown by progressive increase in test line intensity in the LFA as well as a concomitant increase in the percentage of mice showing positive results (**Figure 3**).

### 3.4 LFA performance with mucormycosis patient specimens

**Figure 4:**
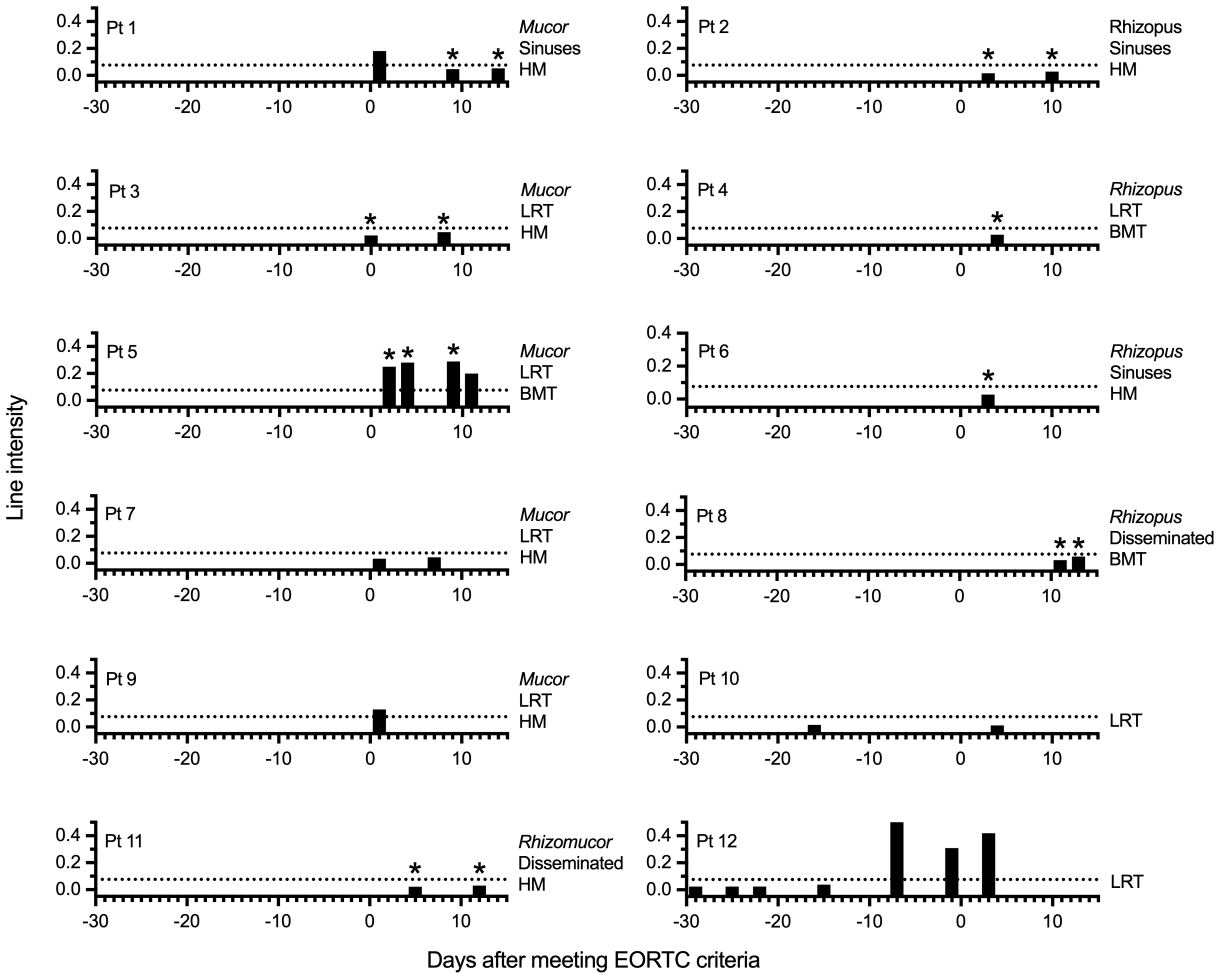
Assay for mannan in sera or plasma of mucormycosis patients. Specimens were collected from individual patients at the indicated times before or after meeting EORTC criteria. When available, patient data including genus, site of infection and predisposing condition (bone marrow transplant or hematologic malignancy) are shown at the right of the immunoassay results. An asterisk above a line intensity bar indicates that the patient was receiving treatment with AmB at the time that the specimen was collected. Patients 10 and 12 were enrolled in the BMT CTN 0101 Trial. All remaining patients were enrolled in the AsTeC study. Dotted horizontal line indicates assay cutoff with serum, which was more conservative (*i.e.* was a higher test line intensity value) than assay cutoff with plasma.

The Mucorales LFA was also evaluated with sera or plasma from 12 patients that met the EORTC criteria for diagnosis of probable or proven invasive mucormycosis and 10 patients with similar high-risk host factors who did not meet the EORTC criteria for invasive fungal disease. Samples were taken from some patients at several times before or after the patients met the EORTC criteria. Four of the 12 patients had positive test results at one or more times (**Figure 4**). With the exception of one patient with consistently high mannan levels, treatment with amphotericin B (AmB) during the time of specimen collection was associated with negative test results. Among the patients for whom specimens were collected while they were not receiving AmB, 4 of the 6 patients had positive test results at one or more times. One patient whose blood was collected prospectively in the BMT CTN 0101 Trial showed a high level of mannan seven days before meeting the EORTC diagnostic criteria. Plasma collected from 10 at-risk but uninfected patients in the AsTeC biorepository were uniformly negative (**Supplementary Table 2**).

## 4 Discussion

This is the first published report of an immunoassay for detection of mannans from the Mucorales in body fluids from animal models of mucormycosis and in patient specimens. There are many reports of shedding of mannans into blood during infection by Ascomycetous fungi, but no such report from the phylogenetically distinct Mucorales. Davies and Thornton reported detection of an extracellular Mucorales polysaccharide with a competitive lateral-flow device (Davies and Thornton, 2022); however, it was not reported whether the detected antigen was a mannan. Moreover, the study was limited to serum spiked with polysaccharide. Unfortunately, little is known about the structure of Mucorales mannan.

Previous studies of mannan immunoassays for diagnosis of invasive fungal disease found that the clinical sensitivity of the immunoassay was influenced by the analytical sensitivity of the test. Increased sensitivity can be achieved by reducing the cutoff values for the test or by using enhanced assay procedures such as biotinyation amplification in ELISA (Mercier et al., 2021; Maertens et al., 2007; Durkin et al., 2008). All mannan immunoassays in current use have the ELISA format or LFAs employing colloidal gold as the indicator particles. A fluorescent reporter particle coupled with an appropriate reader can achieve improvements in sensitivity ranging from 7-100-fold (Juntunen et al., 2012; Xia et al., 2009; Walish et al., 2022). To that end, our europium-based LFA achieved analytical LoDs for members of the Mucorales that ranged from 0.12 to 1.2 ng/ml, depending on the fungal genus and species (**Table 1**).

The Mucorales LFA was highly reactive with mannans from *Rhizopus*, *Mucor*, *Cunninghamella* and *Apophysomyces*, which represent four of the five genera of the Mucorales fungi (**Table 1**) (Chibucos et al., 2016). There was no reactivity with mannan from *Lichtheimia* spp. Notably, *Lichtheimia* isolates are in a separate clade from the other Mucorales (Chibucos et al., 2016). *Lichtheimia* spp. account for approximately 5% of cases of mucormycosis (Roden et al., 2005), but the frequency may be higher in Europe (Gouzien et al., 2024) and lower in India (Prakash et al., 2019), which means that a negative LFA result does not exclude mucormycosis caused by *Lichtheimia*. The Mucorales LFA produced negative results with purified mannans from other fungi that may produce invasive fungal disease, including *Aspergillus*, *Candida* and *Fusarium* (**Table 2**). Further specificity testing showed no reactivity with mannans from a broad spectrum of ascomycetous fungi that are not normally found in IFD. Strong positive results were found with mannans from *Trichophyton*, which is a dermatophyte that causes nail, skin and hair infection, and *Alternaria*, which is a melanized mold that can cause invasive fungal disease (*e.g.* a retrospective review of non-*Aspergillus* and non-Mucorales invasive fungal disease cases identified 11 instances of invasive *Alternaria* infection in a large tertiary-care center in Europe from 1997-2023 (Ledoux et al., 2024)). Neither of these fungi are considered typical causes of invasive fungal disease. The structural basis for this cross-reactivity is not known.

In this study, experiments from a mouse model of pulmonary mucormycosis provided proof-of-concept that mannan in BALF and serum is a biomarker for infection. Levels of mannan in both specimens increased with time after infection. Mannan appeared earliest in BALF, followed by serum, a result expected considering that the primary target organ in this model is the lung. Also, this result is similar to results from studies of galactomannan in serum and BALF in invasive aspergillosis. For example, Nguyen et al. found that testing of BALF galactomannan was more sensitive than cytology, BALF culture, transbronchial biopsy or serum GM for diagnosis of invasive pulmonary aspergillosis in patients with hematologic malignancies and stem cell transplant recipients (Nguyen et al., 2011).

Finally, as a proof-of-concept exploratory study, the mucormycosis LFA was evaluated with serum/plasma from a limited number of patients with mucormycosis; a comparable set of BALF mucormycosis patient specimens was not available for evaluation. Most of these specimens were obtained from the AsTeC specimen repository. The repository is a collection of specimens from patients at high risk for invasive aspergillosis. However, the patient enrollment criteria led to collection of specimens from patients ultimately found to have mucormycosis (Wingard et al., 2021). Specimens from two additional patients were provided from a prospective study of hematopoietic cell transplant patients enrolled in a trial of antifungal prophylaxis (Wingard et al., 2010). Overall, positive test results were found at one or more timepoints from 4 of 12 mucormycosis patients. Notably, serum/plasma tested negative in 7 of 8 patients for whom specimens were collected while they were receiving AmB. This impact of AmB treatment likely accounted for the negative results with specimens from six of the eight patients who tested negative at all timepoints analyzed. A similar result has been observed in use of galactomannan for diagnosis of invasive aspergillosis where sensitivity of GM immunoassay is significantly lower in patients simultaneously receiving antifungal agents (Leeflang et al., 2015; Fisher et al., 2021; Duarte et al., 2014). This suggests that the mucormycosis LFA may be useful as a treatment response indicator as well as a diagnostic.

Traditional tools available for diagnosis of mucormycosis include radiology, microscopy and histology, and culture. More recently, plasma cell-free DNA PCR has shown great promise for non-invasive diagnosis of mucormycosis (Millon et al., 2013, 2022; Mah et al., 2025; Moreno et al., 2024). Such lab-developed tests (LDTs) can enable simultaneous, species-level readout for multiple pathogens (bacterial, viral, and fungal), albeit with concomitant requirements for high-level laboratory infrastructure, personnel expertise, and variable turnaround times. Our results suggest that a LFA for Mucorales is a potential addition to the toolbox in cases where rapid, targeted testing for mucormycosis is needed, especially in clinics without on-site access to a high-complexity reference lab. Use of a europium label produced an LFA with high analytical sensitivity. The LFA strips can be visually inspected by use of a UV penlight or a reader. The Halo reader has the advantages of very low cost and generation of quantitative results (Walish et al., 2022). With the wide use of the LFA format for uses such as pregnancy testing and the COVID-19 diagnostic test, this is a familiar technology that requires little or no training and would be widely accepted. The LFA platform typically generates results in 15–20 min, which means that a mucormycosis immunoassay built in the LFA format could potentially be both low cost and used at or near the point of patient care.

In summary, our results provide proof of concept for use of mannan immunoassay as a strategy for diagnosis of invasive mucormycosis. First, the test detects mannan from four of the five major genera of disease-producing members of the Mucorales. Second, there was no reactivity with mannans from the other major causes of invasive fungal disease, *Aspergillus*, *Candida* and *Fusarium*. Third, the Mucorales LFA detected mannan in serum and BAL fluid from an animal model of invasive mucormycosis. The high levels of mannan in BALF is particularly encouraging because BALF is already commonly used for antigen testing for invasive aspergillosis. A future application in which the same BALF specimen is evaluated for both invasive aspergillosis and mucormycosis would streamline diagnosis in a clinical setting. Finally, the test was positive in sera from 4 of 6 mucormycosis patients who were not simultaneously under treatment with AmB. Taken together, these results demonstrate shedding of mannan during the course of invasive mucormycosis and support future study with a much larger clinical trial (spanning multiple categories of high-risk patients) to determine the extent to which the Mucorales LFA can provide early detection of invasive mucormycosis.

## Data Availability

The raw data supporting the conclusions of this article will be made available by the authors upon request, without undue reservation.
