# Supplementary material for: Mannan immunoassay for diagnosis of invasive mucormycosis: assay development, preclinical testing and evaluation in an animal model
Source: Front Cell Infect Microbiol. 2026 Jul 22;16:1875264. doi: 10.3389/fcimb.2026.1875264 (PMC13437415; doi:10.3389/fcimb.2026.1875264)
Supplement: Supplementary file 1 [file DataSheet1.pdf]

Supplementary Data for:

Mannan immunoassay for diagnosis of invasive mucormycosis: assay development, preclinical testing and evaluation in an animal model

Amanda R. Burnham-Marusich[1*], Vidhi Pandit[1], Sondus Alkhazraji[2], Yiyou Gu[2], Kathleen R. Zayac[1], John R. Wingard[3], Emma L. Culver[4], Joseph J. Walish[4], Ashraf S. Ibrahim[2,5], Thomas R. Kozel[1]

[1]Department of Microbiology and Immunology, University of Nevada, Reno School of Medicine, Reno, NV, USA

[2]The Lundquist Institute, Torrance, CA, USA

[3]Division of Hematology/Oncology, University of Florida College of Medicine, Gainesville, FL, USA

[4]Hyperion Biosystems, Watertown, MA, USA

[5]Division of Infectious Disease, David Geffen School of Medicine, University of California Los Angeles, Los Angeles, CA, USA

**Supplemental Table 1.** Fungi used in study		
Fungus	Strain	Source
Alternaria alternata	6663	ATCC
Apophysomyces elegans	90757	ATCC
Aspergillus fumigatus	MYA-4609	ATCC
Acremonium strictum	Y-48335	NRRL
Candida albicans	MYA-2876	ATCC
Candida auris	CAU-07	CDC
Candida glabrata	48435	ATCC
Candida krusei	90878	ATCC
Candida parapsilosis	22019	ATCC
Cunninghamella bertholletiae	174	a gift from Dr. Thomas Walsh
Cunninghamella bertholletiae	175	A gift from Dr. Thomas Walsh
Fusarium solani	95-2478	a gift from Dr. P. Ferreri
Lichtheimia corymbifera	B2541	Dr. Ashraf Ibrahim lab
Lichtheimia corymbifera	B5792	Dr. Ashraf Ibrahim lab clinical isolate (sputum, 1997, New Mexico, USA)
Mucor circinelloides	8097	ATCC
Mucor circinelloides	8542	ATCC
Penicillium chrysogenum	10106	ATCC
Rhizopus delemar	99-880	Fungal Testing Laboratory, University of Texas Health Sciences Center at San Antonio
Rhizomucor microsporus	FP-469	Ibrahim lab clinical isolate (Birmingham, UK)
Rhizopus oryzae	46599	ATCC
Rhizopus oryzae	99-892	Fungal Testing Laboratory, University of Texas Health Sciences Center at San Antonio
Rhizomucor pusillus	32627	ATCC
Rhizomucor pusillus	46342	ATCC
Scedosporium apiospermum	MYA-3635	ATCC
Trichophyton rubrum	MYA-4438	ATCC

Supplemental Table 2. Results from analysis of serum/plasma from high-risk patients with no fungal infection

Patient ID	Predisposing condition	Line intensity
13	BMT (IA EORTC 263 days after collection date)	0.000
14	BMT	0.000
15	HM (prior to BMT)	0.012
16	HM (prior to BMT)	0.013
17	HM (prior to BMT)	0.016
18	BMT	0.021
19	HM (prior to BMT)	0.014
20	HM (prior to BMT)	0.019
21	HM (prior to BMT)	0.016
22	HM (prior to BMT)	0.011

BMT, bone marrow transplant. IA, invasive aspergillosis. HM, hematological malignancy.
